# MyD88-dependent and independent pathways of Toll-Like Receptors are engaged in biological activity of Triptolide in ligand-stimulated macrophages

**DOI:** 10.1186/1472-6769-10-3

**Published:** 2010-04-12

**Authors:** Vummidigiridhar Premkumar, Moul Dey, Ruth Dorn, Ilya Raskin

**Affiliations:** 1 Cancer and Cell Biology Department, University of Cincinnati, OH 45219, USA; 2 Nutrigenomics Program, South Dakota State University, Brookings, SD 57007, USA; 3 Biotech Center, Rutgers University, 59 Dudley Rd, New Brunswick, NJ 08901, USA

## Abstract

**Background:**

Triptolide is a diterpene triepoxide from the Chinese medicinal plant *Tripterygium wilfordii *Hook F., with known anti-inflammatory, immunosuppressive and anti-cancer properties.

**Results:**

Here we report the expression profile of immune signaling genes modulated by triptolide in LPS induced mouse macrophages. In an array study triptolide treatment modulated expression of 22.5% of one hundred and ninety five immune signaling genes that included Toll-like receptors (TLRs). TLRs elicit immune responses through their coupling with intracellular adaptor molecules, MyD88 and TRIF. Although it is known that triptolide inhibits NFκB activation and other signaling pathways downstream of TLRs, involvement of TLR cascade in triptolide activity was not reported. In this study, we show that triptolide suppresses expression of proinflammatory downstream effectors induced specifically by different TLR agonists. Also, the suppressive effect of triptolide on TLR-induced NFκB activation was observed when either MyD88 or TRIF was knocked out, confirming that both MyD88 and TRIF mediated NFκB activation may be inhibited by triptolide. Within the TLR cascade triptolide downregulates TLR4 and TRIF proteins.

**Conclusions:**

This study reveals involvement of TLR signaling in triptolide activity and further increases understanding of how triptolide activity may downregulate NFκB activation during inflammatory conditions.

## Background

Chronic inflammation is an important patho-physiological condition impacting various diseases including rheumatoid arthritis (RA), atherosclerosis, diabetes, and cancer. Recent evidence suggests the involvement of Toll-like receptors (TLRs) in various chronic inflammatory and autoimmune diseases [1-3]. TLRs belong to the family of pathogen-associated molecular pattern recognition receptors and are vital components of the host's immune system for sensing dangerous pathogens, and for initiating inflammatory and immune responses directed against these pathogens. The mechanism of signal transduction through TLRs is well characterized [4-11]. There are two possible routes for mediation of signals received by TLRs depending on which of the two adapter molecules (MyD88 and TRIF) are involved. The importance of MyD88 and TRIF lies in the finding that each leads to a distinct profile of immune mediators that in turn determine the phenotype of the cells that are primarily responsible for the development of adaptive immune responses [1-4]. TLR4 mediates through both the MyD88 and TRIF pathways, TLR3 signals through TRIF and all the other TLRs mediate through MyD88 pathway [5-8]. Characterization of cellular responses to various ligands that selectively activate specific TLRs is not only useful in better understanding of disease pathogenesis but can also potentially help identify molecular targets by which pharmacological compounds modulate TLR-mediated signaling pathways and target gene expression.

Triptolide is a biologically active diterpene triepoxide from a Chinese herb *Tripterygium wilfordii Hook *F, commonly known as thunder god vine. Extracts from this plant have been historically used in traditional Chinese medicine to treat inflammatory and autoimmune diseases such as rheumatoid arthritis, systemic lupus, psoriatic arthritis and Behcet's disease [12]. Triptolide inhibited the expression of proinflammatory markers including COX-2 and iNOS in RAW macrophage [13]. Triptolide suppressed c-jun NH_2_-terminal kinase (JNK) phosphorylation, COX-2 expression and PGE2 production in microglial cultures treated with lipopolysaccharide [14]. NFκB activation due to inflammatory response in chronic diseases is well characterized [15]. Existing reports demonstrate the effect of triptolide on NFκB activation and target gene expression as well as its effect on other transcription factors [16]. Although TLRs are known to impact downstream NFκB activation [17], effects of triptolide in TLR signaling have not been evaluated. In this study we investigated the effects of triptolide activity on receptors and target gene expression induced by activation of TLRs.

## Methods

### Chemicals and biochemicals

Antibiotics, Dimethyl sulphoxide (DMSO), triptolide (MW 360.4) and LPS (lipopolysaccharide from *E.coli*, serotype 055:B5) were purchased from Sigma chemicals (St. Louis, MO). Ligands for TLR2 (Zymosan) and TLR3 (Poly I:C) were purchased from Invivogen (San Diego, CA). Cell culture media were obtained from Invitrogen Inc. (Carlsbad, CA). Reagents used in quantitative PCR, including enzymes MyD88 and TRIF pre-designed siRNA purchased from Ambion (Austin, TX). RAW 264.7 cell line (ATCC TIB-71) was provided by American Type Culture Collection (Manassas, VA). The BCA Protein Assay kit and NE-PER Extraction kit were obtained from Pierce (Rockford, IL). The ECL Advanced Western blotting detection Kit chemiluminescence system from Amersham Biosciences (Buckinghamshire, UK). Broad ranger blotting markers, anti-TLR4 rabbit polyclonal antibody, anti-actin rabbit polyclonal antibody and Horseradish peroxidase-conjugated anti-rabbit antibody were purchased from Santa Cruz Biotechnology, Inc. (Santa Cruz, CA). Anti-TRIF rabbit polyclonal antibody was purchased from Cell Signaling Technology (Beverly, MA)

### Macrophage cell culture assay

**Figure 1:**
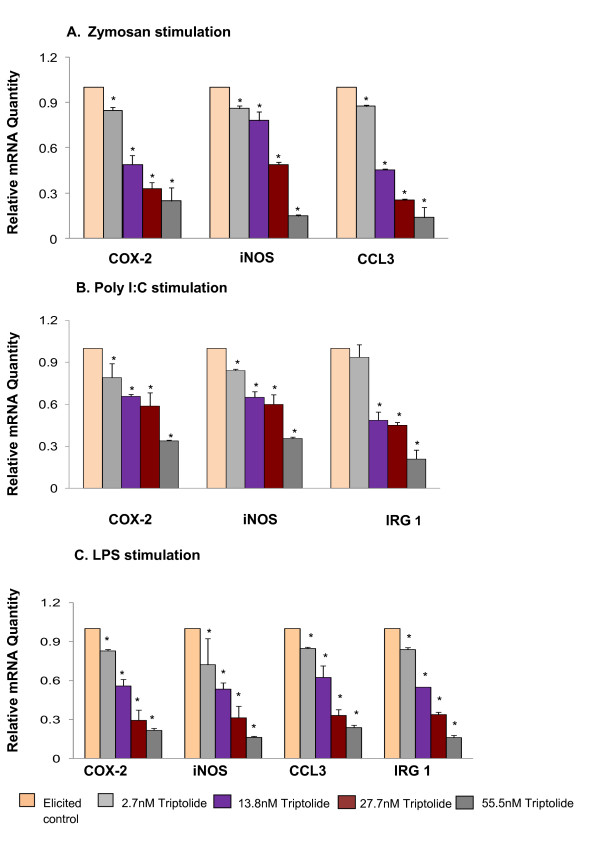
**Effect of triptolide on gene expression of COX-2, iNOS and chemokines in response to various TLR ligand activation in RAW macrophages**. The effect of triptolide treatment (three replicates) on a specific gene expression was measured by the mRNA quantity relative to the response to ligand activation only (positive control) that was normalized to a value of 1.00; lower values represent greater inhibitory effects with 0.00 corresponding to a complete inhibition of the induced gene expression. The value of the negative control (no induction) was normalized to 0.00. Values are mean ± S.D. *, *p *< 0.05; (post-ANOVA comparison with Ligand-treated positive control). A. Effect of triptolide on mRNA levels of COX-2, iNOS and CCL3 following zymosan stimulation. B. Effect of triptolide on mRNA levels of COX-2, iNOS, and IRG-1 following Poly I:C stimulation. C. Effect of triptolide on mRNA levels of COX-2, iNOS, CCL3 and IRG-1 following LPS stimulation.

**Figure 2:**
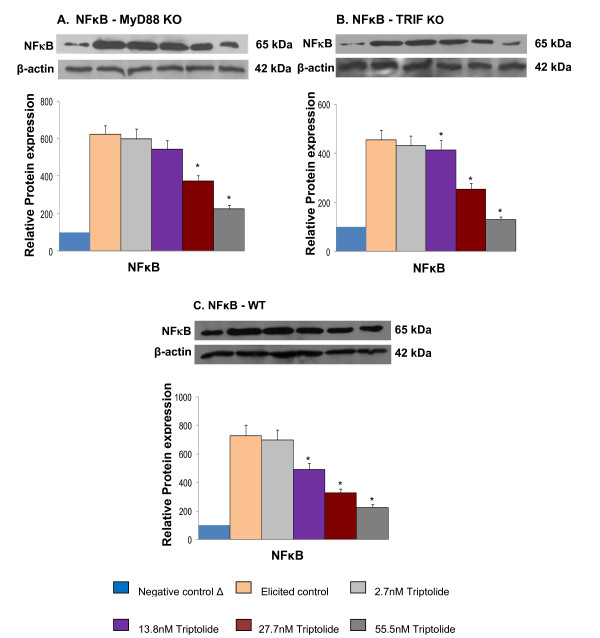
**Effect of triptolide on NFκB p65 nuclear translocation**. Representative immunoblots of NFκB (p65). β-actin used as internal control. The protein expression was measured by densitometric analysis (Total Labs software v 2.01). The untreated control T^- ^was normalized to a value of 100. Each value represents mean ± SD of three experiments performed in triplicate. A. Effect of triptolide on NFκB translocation in MyD88KO RAW macrophages B. Effect of triptolide on NFκB translocation in TRIFKO RAW macrophages C. Effect of triptolide on NFκB translocation in Wild-type RAW macrophages. * Significantly different from control (p < 0.05) ANOVA followed by LSD

**Figure 3:**
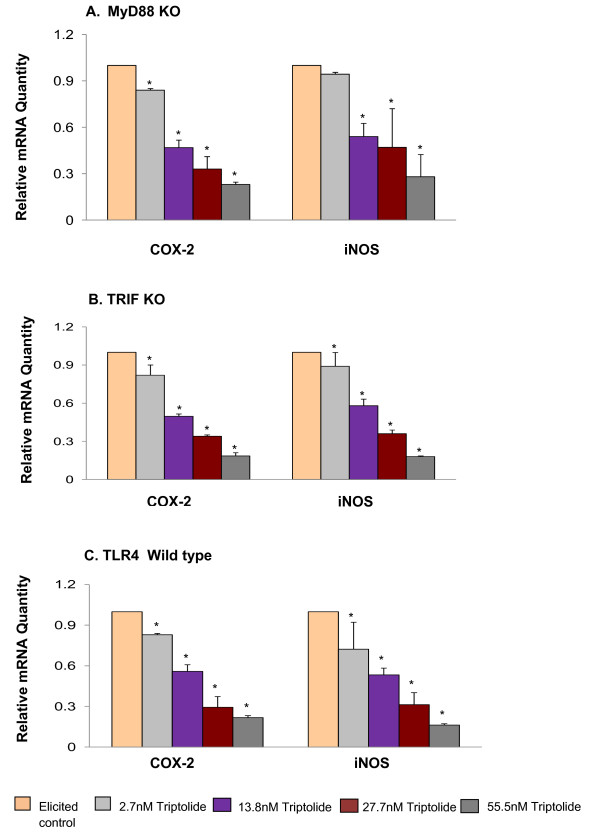
**Effect of triptolide on gene expression of COX-2 and iNOS (mean ± S.D)**. The expression of specific genes was measured by the mRNA quantity relative to the response to LPS activation (positive control) that was normalized to a value of 1.00; lower values represent greater inhibitory effects with 0.00 corresponding to a complete inhibition of the induced gene expression. The value of the negative control (no induction) was normalized to 0.00. A. Expression of COX-2 and iNOS genes in MyD88KO macrophages; B. Expression of COX-2 and iNOS genes in TRIFKO macrophages; C. Expression of COX-2 and iNOS genes in WT macrophages. * Significantly different from control (p < 0.05) ANOVA followed by LSD.

**Figure 4:**
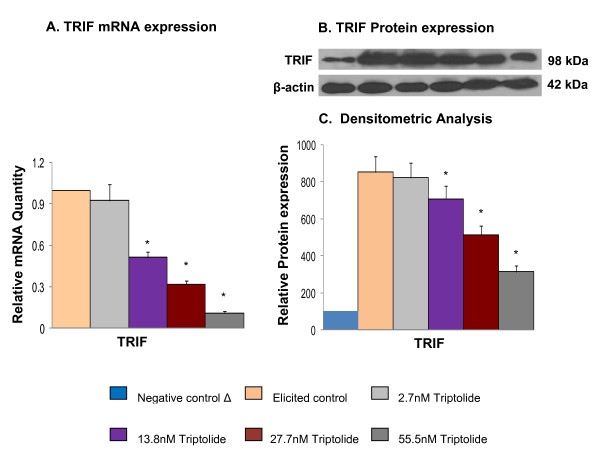
**Effect of triptolide on mRNA and protein expression of TRIF (mean ± S.D)**. The mRNA expression of TRIF was measured by the mRNA quantity relative to the response to LPS activation (positive control) that was normalized to a value of 1.00; lower values represent greater inhibitory effects with 0.00 corresponding to a complete inhibition of the induced gene expression. The value of negative control (no induction) was normalized to 0.00. A. mRNA expression of TRIF; B. Protein expression of TRIF; C. Densitometric analysis for protein expression. * Significantly different from control (p < 0.05) ANOVA followed by LSD.

**Figure 5:**
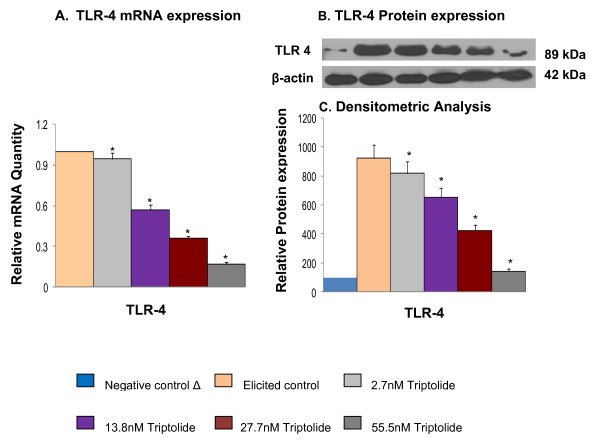
**Effect of triptolide on mRNA and protein expression of TLR 4 (mean ± S.D.)**. The mRNA expression of TLR 4 was measured by the mRNA quantity relative to the response to LPS activation (positive control) that was normalized to a value of 1.00; lower values represent greater inhibitory effects with 0.00 corresponding to a complete inhibition of the induced gene expression. The value of negative control (no induction) was normalized to 0.00. A. mRNA expression of TLR 4; B. Protein expression of TLR 4; C. Densitometric analysis for protein expression. * Significantly different from control (p < 0.05) ANOVA followed by LSD

RAW 264.7 macrophage cells were cultured as described by Dey *et al.*, 2006 [18]. Briefly, cells were seeded at a density of 0.4 × 10^6^cells per well (viable cell counts were carried out by trypan blue staining using a hemocytometer) in 24-well plates 12 h prior to treatment. The cells were then treated with triptolide dissolved in DMSO at concentration (per ml of cells) of 20 ng, 10 ng, 5 ng and 1 ng for 2 h before elicitation with bacterial endotoxin LPS (lipo-polysaccharide from E.coli, serotype 055:B5) at 1 μg/ml for TLR-4 elicitation, Zymosan at 1 ug/ml for TLR-2 elicitation and Poly I:C (synthetic analog of dsRNA) at 10 ug/ml for TLR-3 elicitation. The corresponding molar concentrations of triptolide (MW 360.4) that are used in Figures 1, 2, 3, 4 and 5 were 55.5 nM, 27.7 nM, 13.8 nM and 2.7 nM respectively. For each experiment, one positive control (cells treated with ligands and vehicle) and one negative control (cells treated with vehicle only) were included. For RNA extraction, the cells were harvested in TRIzol after 6 h of ligand stimulation and for protein expression the cells were harvested after 12 h. Two replicates were made for all the treatments and their controls. The concentrations of triptolide used were previously tested to be non-cytotoxic using a MTT assay [13]. The CC_50 _(concentration at which 50% of cells remain viable) of triptolide for macrophages is ~83.2 nM.

### MyD88KO and TRIFKO using RNAi

Pre-designed small interfering RNA (siRNA) oligonucleotides targeting endogenous MyD88 and TRIF were purchased from Ambion (Austin, TX). The siRNA duplexes were transfected using lipofectamine 2000 (Invitrogen) into RAW 264.7 cells following the manufacturer's protocol. Briefly, cells were plated at 0.2 × 10^6 ^cell/well in a 24-well plate maintained in Dulbecco's modified Eagle's medium (DMEM) supplemented with 10% heat-inactivated fetal bovine serum. After 24 h, cells were treated with 1 μl of 50 μM MyD88-siRNA or TRIF-siRNA in a transfection mixture containing lipofectamine 2000 and incubated in 5% CO_2_, at 37°C. After 24 h of transfection the medium was changed with 1 ml of fresh DMEM. Two hours before elicitation with LPS (1 μg/mL), the cells were treated with predetermined doses of triptolide. For RNA extraction, the cells were harvested in TRIzol after 6 h of treatment and for protein expression the cells were harvested after 12 h.

### Total RNA extraction, purification, and cDNA synthesis

Total RNA extraction, purification and cDNA synthesis were performed following procedures described in Dey *et al.*, 2006 [18].

### Quantitative polymerase chain reaction and gene array

qRT-PCR was performed as described by Dey *et al.*, 2006 [18]. Gene-specific primers (synthesized by IDT Inc., Coralville, IA) used in the current study are described in Table 1. For the gene array experiment, PCR-arrays (APM_025, SABiosciences, MD) were purchased and the manufacturer's protocol was followed. Relative quantification based on SYBR green was used for individual and gene array experiments.

**Table 1:** Sequence of primers used for real time RT-PCR.

Gene symbol* (accession#)	Forward primer	Reverse primer
β-actin (NM_007393)	5'AACCGTGAAAAGATGACCCAGAT3'	5'CACAGCCTGGATGGCTACGT3'
COX-2 (NM_011198)	5'TGGTGCCTGGTCTGATGATG3'	5'GTGGTAACCGCTCAGGTGTTG3'
iNOS (XM_147149)	5'CCCTCCTGATCTTGTGTTGGA3'	5'TCAACCCGAGCTCCTGGAA3'
MyD88 (NM_010851)	5'TGGCCTTGTTAGACCGTGA3'	5'AAGTATTTCTGGCAGTCCTCCTC3'
TRIF (NM_174989)	5'TGGCAAACACCTTCAAGACA3'	5'GCGCTTTCTTCCAGCGTA3'
CCL3 (NM_011337)	5'TGCCCTTGCTGTTCTTCTCT3'	5'GTGGAATCTTCCGGCTGTAG3'
IRG1 (NM_008392)	5'GCTTTTGTTAATGGTGTTGCTG3'	5'GGCTTCCGATAGAGCTGTGA3'
TLR2 (NM_011905)	5'GGGGCTTCACTTCTCTGCTT3'	5'AGCATCCTCTGAGATTTGACG3'
TLR3 (NM_126166)	5'GATACAGGGATTGCACCCATA3'	5'TCCCCCAAAGGAGTACATTAGA3'
TLR4 (NM_021297)	5'GGACTCTGATCATGGCACTG3'	5'CTGATCCATGCATTGGTAGGT3'

*Refer to abbreviation list for full gene names

### Immunoblotting analysis of TRIF and TLR4

The cells were lysed using RIPA buffer (Pierce, Rockford, IL) according to the manufacturer's protocol. Equal amounts of total cellular protein (20 μg) was quantified using BCA protein assay kit according to the kit's protocol. The samples were resolved by 10% SDS-PAGE under reducing conditions (100 V, 2 h) and transferred to nitrocellulose membranes (50 V, 2 h) in a buffer consisting of 20% v/v methanol, 200 mM Glycine, 25 mM Tris, pH 8.3. The membrane was blocked for overnight at 4°C and then incubated with anti-TRIF rabbit polyclonal antibody (1:1000) or anti-TLR4 rabbit polyclonal antibody (1:1000) or anti-actin rabbit polyclonal antibody (1:5000) for 2 h at RT. Horseradish peroxidase-conjugated secondary anti-rabbit antibody was used and incubated for 1 h at RT. Immunodetection was performed using an ECL Advanced Western blotting detection Kit chemiluminescence system. The autoradiograms were quantified using scanning densitometry (Total Labs software v 2.01).

### Cell fractionation and transactivation of NFκB (p65)

Nuclear extracts were prepared according to the instructions provided in NE-PER™ Pierce Nuclear and Cytoplasmic extraction kit). Cells were collected 45 minutes after LPS induction (1 μg/ml). A 50 μg amount of nuclear extracts from macrophage cells were electrophoresed in 12% SDS-PAGE under reducing conditions, transferred to nitrocellulose membranes and blocked with 5% Non-fat milk powder in PBS. Membranes were incubated with rabbit polyclonal antibodies to p65 (1/500) or anti-actin. Secondary anti-rabbit peroxidase bound antibody was used. The immunodetection was performed using an ECL Advanced western blotting detection Kit chemiluminescence system. The autoradiograms were quantified using scanning densitometry (Total Labs software v 2.01).

### Statistical analysis

The data are expressed as Mean ± Standard Deviation (SD). Statistical significance for the data for mRNA and densitometric analysis were calculated using analysis of variance (ANOVA) and the group means were compared by the least significant difference test (LSD). The results were considered statistically significant if *p *< 0.05.

## Results

### Gene array data confirmed known and revealed unknown genes affected by triptolide

Expression of one hundred and ninety five target genes in response to triptolide treatment were studied by gene array in LPS stimulated mouse macrophages. The genes based on their response to LPS stimulation were characterized as LPS-responsive (Table 2) and LPS-nonresponsive genes (Table 3). Among LPS-responsive genes, 42 genes were downregulated (Table 2a) and 2 genes (Table 2b) were upregulated by triptolide treatment. Fourteen genes were found to be non-responsive to LPS induction (Table 3). Of these non-responsive genes, 8 genes were downregulated (Table 3a) and 6 genes were upregulated by triptolide treatment (Table 3b). Huang *et al. *(2006) reported that 320 genes were upregulated in RAW 264.7 cells in response to LPS treatment but only 32 (10%) genes were downregulated by triptolide [19]. In our study that included some overlapping genes with Huang *et al *(2006) [19], triptolide down regulated 21% of the LPS-induced genes and affected a total of 22.5% of LPS-responsive genes (including down and upregulations) in RAW macrophages. Triptolide treatment was found to downregulate the expression of TLRs 1 and 4 (Table 2a), TLRs 3 and 7 (Table 3a), TNF, IL-6, IL-1, NFκB1, MAPK, Rel, Bcl3, COX-2 (Table 2a) and other important inflammation regulating genes (Tables 2, 3, 4). The gene array results obtained for COX-2, TNFα, IL1β (data not shown) and TLR4 were further validated using qRT-PCR. We further investigated expression of TLR-mediated genes to understand in greater details the extent of involvement of TLR signaling cascade in the activity of triptolide.

**Table 2:** Fold changes in gene expression in cells treated with triptolide (55.5 nM)+LPS (1 μg/ml) as compared to those treated with LPS (1 μg/ml) alone.

a) Downregulation of LPS- stimulated genes by triptolide.				
**Gene**	**Accession No.**	**Gene name**	**Fold change in response to treatment**	

**Symbol**			LPS*	**LPS+triptolide**

Ccl2	NM_011333	Chemokine (C-C motif) ligand 2	19.90	-11.98
Ccl22	NM_011331	Chemokine (C-C motif) ligand 12	692.18	-8.53
Ccl3	NM_011337	Chemokine (C-C motif) ligand 3	4.48	-3.82
Ccl5	NM_013653	Chemokine (C-C motif) ligand 5	22.86	-19.05
Ccl7	NM_013654	Chemokine (C-C motif) ligand 7	96.67	-19.73
Ccl9	NM_011338	Chemokine (C-C motif) ligand 9	77.98	-30.74
Cxcl10	NM_021274	Chemokine (C-X-C motif) ligand 10	113.38	-39.18
Cxcl11	NM_019494	Chemokine (C-X-C motif) ligand 11	41.50	-13.01
Il10	NM_010548	Interleukin 10	55.52	-47.24
Il10ra	NM_008348	Interleukin 10 receptor, alpha	4.01	-3.21
Il13ra1	NM_133990	Interleukin 13 receptor, alpha 1	13.13	-11.17
Il18	NM_008360	Interleukin 18	17.33	-7.68
Il1a	NM_010554	Interleukin 1 alpha	2360.70	-102.68
Il1b	NM_008361	Interleukin 1 beta	1140.14	-55.02
Il1f6	NM_019450	Interleukin 1 family, member 6	51.09	-39.18
Il2rg	NM_013563	Interleukin 2 receptor, gamma chain	15.73	-5.66
Itgam	NM_008401	Integrin alpha M	7.09	-5.91
Itgb2	NM_008404	Integrin beta 2	3.82	-4.63
Spp1	NM_009263	Secreted phosphoprotein 1	5.60	-3.25
Tgfb1	NM_011577	Transforming growth factor, beta 1	4.27	-1161.68
Tnf	NM_013693	Tumor necrosis factor	58.69	-45.32
Tnfrsf1b	NM_011610	Tumor necrosis factor receptor superfamily, member 1b	90.82	-16.94
Bcl3	NM_033601	B-cell leukemia/lymphoma 3	20.51	-20.30
Crebbp	NM_001025432	CREB binding protein	2.92	-4.33
Csf2	NM_009969	Colony stimulating factor 2 (granulocyte-macrophage)	116.81	-61.53
Csf3	NM_009971	Colony stimulating factor 3 (granulocyte)	13568.7	-355.41
Gja1	NM_010288	Gap junction membrane channel protein alpha 1	4.65	-6.20
Il6	NM_031168	Interleukin 6	674.65	-256.59
Nfkb1	NM_008689	Nuclear factor of kappa light chain gene enhancer in B-cells 1, p105	32.63	-3.37
Nfkbia	NM_010907	Nuclear factor of kappa light chain gene enhancer in B-cells inhibitor, alpha	18.87	-2.93
Rel	NM_009044	Reticuloendotheliosis oncogene	114.40	-14.86
Ripk1	NM_009068	Receptor (TNFRSF)-interacting serine-threonine kinase 1	3.18	-7.80
Tlr1	NM_030682	Toll-like receptor 1	43.35	-18.55
Tlr4	NM_021297	Toll-like receptor 4	3.27	-5.40
Tnfaip3	NM_009397	Tumor necrosis factor, alpha-induced protein 3	56.41	-5.15
Cd14	NM_009841	CD14 antigen	4.01	-7.56
Cd86	NM_019388	CD86 antigen	4.24	-2.90
Ifnb1	NM_010510	Interferon beta 1, fibroblast	5.56	-7.15
Irf3	NM_016849	Interferon regulatory factor 3	3.02	-58.79
Ly86	NM_010745	Lymphocyte antigen 86	5.15	-8.21
Mapk8	NM_016700	Mitogen activated protein kinase 8	5.68	-11.06
Ptgs2	NM_011198	Prostaglandin-endoperoxide synthase 2	566.13	-51.89

**b) Upregulation of LPS down regulated genes by triptolide.**				

**Gene symbol**	**Accession No.**	**Gene Name**	**Fold change in response to treatment**	
			LPS*	**LPS+triptolide**

Smad3	NM_016769	MAD homolog 3 (Drosophila)	-13.20	4.02
Mapk8ip3	NM_013931	Mitogen-activated protein kinase 8 interacting protein 3	-5.19	4.21

* Fold changes for LPS treatments were determined by comparing to base level expressions in untreated cells

Positive values (≥+3) indicate stimulation while negative values (≤-3) represent suppression of gene expression.

**Table 3:** Fold changes in gene expression in cells treated with triptolide (55.5 nM) as compared to endogenous expression levels in untreated cells.

a) Downregulation (≤-3) of LPS-non responsive genes by triptolide.			
**Gene symbol**	**Accession No.**	**Gene Name**	**Fold change in response to triptolide**

Bcl6	NM_009744	B-cell leukemia/lymphoma 6	-5.36
Cxcr3	NM_009910	Chemokine (C-X-C motif) receptor 3	-7.63
Tnfrsf1a	NM_011609	Tumor necrosis factor receptor superfamily, member 1a	-5.18
Eif2ak2	NM_011163	Eukaryotic translation initiation factor 2-alpha kinase 2	-4.51
Tlr3	NM_126166	Toll-like receptor 3	-10.73
Tlr7	NM_133211	Toll-like receptor 7	-12.85
Ly96	NM_016923	Lymphocyte antigen 96	-4.28
Map2k4	NM_009157	Mitogen activated protein kinase kinase 4	-3.01

**b) Upregulation (≥+3) of LPS-non responsive genes by triptolide.**			

**Gene symbol**	**Accession No.**	**Gene Name**	**Fold change in response to triptolide**

Ccl25	NM_009138	Chemokine (C-C motif) ligand 25	6.05
Fasl	NM_010177	Fas ligand (TNF superfamily, member 6)	115.09
Irf1	NM_008390	Interferon regulatory factor 1	7.60
Mapk3	NM_011952	Mitogen activated protein kinase 3	5.84
Tnfsf14	NM_019418	Tumor necrosis factor (ligand) superfamily, member 14	17.71
Traf3	NM_011632	Tnf receptor-associated factor 3	3.38

LPS (1 μg/ml) did not have any effect on these genes.

**Table 4:** Fold changes in gene expression in cells treated with LPS (1 μg/ml) as compared to endogenous expression levels in untreated cells.

Gene symbol	Accession No.	Gene name	Fold change in response to LPS
C3	NM_009778	Complement component 3	3.02
Il15	NM_008357	Interleukin 15	6.39
Ltb	NM_008518	Lymphotoxin B	4.48
Atf1	NM_007497	Activating transcription factor 1	4.43
Bcl10	NM_009740	B-cell leukemia/lymphoma 10	22.13
Cflar	NM_009805	CASP8 and FADD-like apoptosis regulator	14.01
Edg2	NM_010336	Endothelial differentiation, lysophosphatidic acid G-protein-coupled receptor, 2	6.49
Relb	NM_009046	Avian reticuloendotheliosis viral (v-rel) oncogene related B	21.38
Stat1	NM_009283	Signal transducer and activator of transcription 1	5.89
Cd40	NM_011611	Cluster of Differentiation 40	112.83
Nr2c2	NM_011630	Nuclear receptor subfamily 2, group C, member 2	-4.07
Peli1	NM_030015	Pellino 1	5.48

Triptolide (55.5 nM) did not have any effect on these genes.

### Effect of triptolide on downstream effector expression induced by TLR ligands

There are three possible routes through which TLR signaling is mediated [5-8]. These routes either involve the adapter molecule MyD88, TRIF or both. TLR3 signals through TRIF and all other TLRs mediate through MyD88 pathway whereas TLR4 utilizes both MyD88 and TRIF to transduce the signal it receives [5-8]. One of the important readouts of TLR ligand induction is a robust pro-inflammatory response such as an upregulated expression/secretion of chemokines and cytokines. Using this modulation of chemokines/cytokine expression during different ligand induction the response to triptolide treatment was evaluated. Triptolide inhibited the expression of COX-2 and iNOS induced by MyD88- specific ligand - Zymosan (TLR2), TRIF- specific ligand, Poly I:C (TLR3) and LPS (TLR4) which activates both MyD88 and TRIF pathways (Figure 1). Downregulation of COX-2 and iNOS by triptolide [13] along with selected cytokines/chemokines that are specific to each route of TLR signaling have been used to validate downstream effects of triptolide along the TLR pathway. COX-2 and iNOS expression is common to both MyD88-dependent and -independent (TRIF) signaling pathways. The expression of CCL3 is specific for MyD88-dependent pathway and expression of IRG-1 is a specific readout of the TRIF-dependent pathway [20-22]. Triptolide inhibited the expression of CCL3 in macrophage induced with Zymosan (MyD88 specific ligand) and LPS (TLR4 specific but utilizes both TRIF and MyD88) (Figure 1A and Figure 1C). Triptolide also inhibited the expression of IRG-1 in macrophages induced with Poly I:C (TRIF specific ligand) and LPS (Figure 1B and Figure 1C). These results demonstrate that triptolide inhibits the activation of chemokines involved in both MyD88 and TRIF dependent pathways.

### Effect of triptolide on MyD88KO and TRIFKO in RAW cells induced with LPS

To further validate the effect of triptolide on inflammatory pathways of MyD88 and TRIF, we evaluated the effect of triptolide on LPS induced macrophage under the two following scenarios: (1) MyD88 mediated pathway in the absence of TRIF regulation and (2) TRIF mediated pathway in the absence of MyD88 regulation. As both MyD88 and TRIF signaling pathways lead to NFκB activation [8], we determined the activity of triptolide on NFκB translocation to the nucleus (Figure 2). Also, because COX-2 and iNOS are downstream target genes regulated by NFκB, their increased expression is an indirect indicator of NFκB activation. To further validate results of Figure 1, whether or not triptolide modulates both MyD88 and TRIF mediated signaling pathways, the activation of NFκB and the expression of COX-2 and iNOS induced by LPS in MYD88-KO macrophages and TRIF-KO macrophages were determined. Triptolide suppressed LPS-induced NFκB translocation in a dose dependent manner as determined by immunoblotting of the p65 protein in nuclear extracts. We observed that the incubation of MyD88-KO and TRIF-KO macrophages with LPS (1 μg/mL) produced an increase in NFκB translocation (p65 subunit) to the nuclear compartment that was evident at 45 mins of incubation time. Figure 2A, 2B and 2C show that this translocation process was inhibited in MyD88-KO, TRIF-KO and Wild-type macrophages by pretreatment with triptolide in a dose dependent manner. Similarly, triptolide inhibited the gene expression of COX-2 and iNOS induced by LPS in MyD88-KO, TRIF-KO and Wild-type macrophages in a dose dependent manner (Figure 3A, 3B and 3C). Together, these results demonstrate that triptolide suppresses both MyD88 and TRIF -dependent signaling pathways.

### Effect of triptolide activity on protein and mRNA expression of adaptor molecules and TLR

Triptolide was found to suppress both MyD88 and TRIF-dependent signaling pathways activated by LPS agonization of TLR4. (Figures 2, 3). Therefore, to further characterize the role of triptolide in the signaling events triggered by LPS in macrophages upstream of NFκB, we studied the expression of adapter molecules TRIF and MyD88 as well as that of TLR4 receptor at mRNA and protein levels. Triptolide treatment suppressed the mRNA and protein levels of LPS-induced TLR4 (Figure 5) and TRIF (Figure 4) expression but not MyD88 (data not shown). Suppression of poly I:C induced TLR3 mRNA expression by triptolide, the only TRIF specific toll-like receptor, has not been observed by qRT-PCR (data not shown). Together these observations suggest that triptolide inhibition of NFκB may be directed from the receptor level for MyD88 pathway and from the adapter level for TRIF pathway.

## Discussion

Past studies have demonstrated that triptolide can induce anti-inflammatory responses in several assay systems [14,23]. However, the direct molecular targets of triptolide have remained elusive. In our previous report, we showed that triptolide significantly inhibited the secretion of inflammatory cytokines that occurred with RAW 264.7 cells when stimulated with LPS [13]. Subsequently, we performed a gene array analysis (Tables 2, 3, 4) to elucidate additional members of the immune signaling cascade that could be potential targets of triptolide activity. The results of this analysis showed that 44 genes, which were known to regulate inflammation were differentially expressed in stimulated RAW 264.7 cells following triptolide treatment. These 44 genes represented key immune signaling pathways such as TLR signaling, MAPK signaling, Jak-STAT signaling and cytokine-cytokine receptor interaction. In our present study we undertook further investigation on cellular mechanism of triptolide activity that focused on TLRs, given their upstream location in the immune signaling cascade and their increasingly recognized importance in inflammatory and autoimmune diseases [24].

LPS induces TLR4 dimerization to trigger the activation of downstream signaling pathways [25,26]. This receptor dimerization activates transcription factor NFκB, leading to the induction of inflammatory gene products such as COX-2 and iNOS [25,26]. Many studies have demonstrated that triptolide and its synthetic derivatives inhibited NFκB activation induced by TLR4 agonist LPS [13,14,27] but effect of triptolide on any TLR expression was never reported. The present study shows that triptolide suppressed ligand (LPS)-induced expression of TLR4 at mRNA and protein levels (Figure 5). We also observed for the first time that triptolide suppressed Poly(I:C) (TLR3 agonist) and Zymosan (TLR2 agonist) induced expression of COX-2 and iNOS (Figure 1A, B). These observations suggest that triptolide may offer protection against wide range of infections that occurs by different TLR inductions.

The TLR-ligand activities are transduced through specific intracellular adaptor molecules, most notably MyD88 and TRIF. The importance of MyD88 and TRIF lies in finding that each leads to a distinct profile of immune mediators that in turn determines the phenotype of the cells that primarily are responsible for the development of adaptive immune responses [1-4]. By studying the expression of well-characterized cytokine/chemokine target genes downstream of MyD88 (e.g., CCL3) and TRIF (e.g., IRG-1) [21,22] we demonstrated differential regulation exerted by triptolide in ligand-induced 264.7 RAW macrophages. We observed that triptolide downregulated the expression of both MyD88-dependent cytokine such as CCL3 induced by MyD88-dependent ligands (Zymosan/TLR2 ligand, LPS/TLR4) and TRIF-dependent cytokine IRG-1 (Poly I:C/TLR3 and LPS/TLR4) in a concentration dependent manner (Figure 1A, 1B and 1C). To further confirm that both MyD88 and TRIF mediated signaling are involved in triptolide activity, we showed that triptolide suppressed the NFκB translocation (p65 molecule) to the nucleus and also the downstream expression of COX-2 and iNOS mRNA in MyD88 KO (when only TRIF is present) and TRIF-KO (when only MyD88 is present) macrophages as well as in wild-type macrophages induced with TLR specific ligand, LPS (Figure 2 and 3). These results show that inhibition of NFκB activation and COX-2 and iNOS expression by triptolide could be achieved in presence of MyD88 or TRIF alone or when both are present as in wild type macrophages. These results also suggest that triptolide can block both MyD88- and TRIF-dependent pathways that lead to NFκB transactivation. Since MyD88 and TRIF are the only adapters exclusively mediating all TLR signaling, it is possible that triptolide may suppress wide-range of TLR signaling through other TLRs in addition to TLR4. Although in the current study we focused on TLR4 pathway, our gene array data (Tables 2a and 3a) as well as the observations presented in Figure 1A and 1B may support the suggestion that triptolide has activities against other TLRs. When the changes in the expression of TRIF and MyD88 in response to triptolide were tested (Figure 4), triptolide inhibited the mRNA and protein expression of TRIF, but not MyD88 in a dose dependent manner. A study by Yamamoto *et al. *(2003) [28] using TRIF knockout mice has revealed that TRIF is physiologically essential for TLR-3 mediated signaling and that TRIF is involved in the LPS-induced MyD88-independent pathway.

Triptolide blocked early signaling pathways of TLRs suggesting that its inhibitory action was at the receptor and adapter molecule levels. Thus, a direct interaction of triptolide with TLR4 may be hypothesized. This study suggests that plant compounds, such as triptolide, can modulate TLR-mediated inflammatory responses and can reduce the risk of chronic diseases, associated with exaggerated TLR activation. Macrophages are the key antigen presenting cells in the pathogenesis of RA and involvement of TLR4 has been shown to play role in joint destruction in RA [29]. Therefore, the present study showing interaction of triptolide with components of TLR signaling, such as TLR4, are particularly relevant and support the recent promise shown in clinics against RA by botanical extracts containing triptolide [30,31].

## Conclusions

The results from the present study suggest that the suppression of agonist-induced NFκB activation and chemokine expression by triptolide is mediated by targeting the early signaling of TLRs, particularly that of TLR4 in RAW 264.7 cells. Triptolide downregulated the expression of TLR4 proteins and that of TRIF adapter proteins in the MyD88-independent pathway of TLR4. In addition gene expression profiles in response to triptolide treatment in stimulated macrophages suggest that triptolide may have multiple cellular targets contributing to its strong anti-inflammatory and immune suppressive properties.

## Abbreviations

COX-2: Cycloxygenase-2; iNOS: inducible Nitric oxide synthase; IRG: Interferon regulating gene; LPS: Lipopolysaccharide; MyD88: Myeloid differentiation 88; NFκB: Nuclear factor kappa B; Poly I:C: polyinosinic:polycytidylic acid; TLRs: Toll like Receptors; TRIF: TIR-domain-containing adapter-inducing interferon-β; CCL3: Chemokine (C-C motif) ligand 3.

## Authors' contributions

MD conceived of the study, designed all experiments, carried out, analyzed and interpreted gene array experiments and data. MD also wrote part of the manuscript. VGP carried out rest of the experiments, analyzed his part of data and helped with drafting the manuscript. VGP also interpreted his analyzed data in consultation with MD. RD participated in real-time PCR experiments and helped in proof reading the manuscript for English grammar. IR provided facilities within his laboratory for all experiments at Rutgers University. IR also provided critical comments on the manuscript. All authors read and approved the final manuscript.

## Author information

MD, VGP and IR have a Ph.D in Biology or related fields as their highest obtained degree. RD has a M.S. in Horticulture and is currently studying towards her MBA. MD was formerly an Assistant Research Professor at the Biotechnology Center at Rutgers University, NJ and currently an Associate Professor in the Nutrigenomics Program, South Dakota state University, SD. MD specializes in Molecular Biology. VGP was formerly a Postdoctoral Associate at Rutgers University and is currently working as a Postdoctoral Researcher at University of Cincinnati, OH. RD is a Senior Laboratory Technician in IR's group at Rutgers University, NJ. IR is a Professor in the Department of Plant Biology and Pathology at Rutgers University, NJ.
